# Naturalness in low-scale SUSY models and “non-linear” MSSM

**DOI:** 10.1140/epjc/s10052-014-3050-9

**Published:** 2014-09-25

**Authors:** I. Antoniadis, E. M. Babalic, D. M. Ghilencea

**Affiliations:** 1 CERN Theory Division, 1211 Geneva 23, Switzerland; 2 Theoretical Physics Department, National Institute of Physics and Nuclear Engineering (IFIN-HH), MG-6, 077125 Bucharest, Romania; 3 Department of Mathematics and Natural Sciences, University of Craiova, 13 A. I., Cuza street, 200585 Craiova, Romania

## Abstract

In MSSM models with various boundary conditions for the soft breaking terms ($m_\mathrm{soft}$) and for a Higgs mass of 126 GeV, there is a (minimal) electroweak fine-tuning $\Delta \approx 800$ to $1000$ for the constrained MSSM and $\Delta \approx 500$ for non-universal gaugino masses. These values, often regarded as unacceptably large, may indicate a problem of supersymmetry (SUSY) breaking, rather than of SUSY itself. A minimal modification of these models is to lower the SUSY breaking scale in the hidden sector ($\sqrt{f}$) to few TeV, which we show to restore naturalness to more acceptable levels $\Delta \approx 80$ for the most conservative case of low $\tan \beta$ and ultraviolet boundary conditions as in the constrained MSSM. This is done without introducing additional fields in the visible sector, unlike other models that attempt to reduce $\Delta$. In the present case $\Delta$ is reduced due to additional (effective) quartic Higgs couplings proportional to the ratio $m_\mathrm{soft}/\sqrt{f}$ of the visible to the hidden sector SUSY breaking scales. These couplings are generated by the auxiliary component of the goldstino superfield. The model is discussed in the limit its sgoldstino component is integrated out so this superfield is realized non-linearly (hence the name of the model) while the other MSSM superfields are in their linear realization. By increasing the hidden sector scale $\sqrt{f}$ one obtains a continuous transition for fine-tuning values, from this model to the usual (gravity mediated) MSSM-like models.

## Introduction

If supersymmetry (SUSY) is realized in Nature, it should be broken at some high scale. A consequence of SUSY breaking is the existence of a Goldstone fermion—the goldstino—and its scalar superpartner, the sgoldstino. The goldstino becomes the longitudinal component of the gravitino which is rendered massive (super-Higgs mechanism), with a mass of order $f/M_P$ where $\sqrt{f}$ is the scale of spontaneous supersymmetry breaking in the hidden sector and $M_P$ is the Planck scale. Also, the sgoldstino can become massive and decouple at low energies. One interesting possibility is that $\sqrt{f}\ll M_P$, which represents the case of the so-called low-scale SUSY breaking models that we analyze in this work. Then the longitudinal gravitino component couplings which are those of the goldstino and proportional to $1/\sqrt{f}$ [1–5] are much stronger than the couplings of the transverse gravitino component fields, which are Planck-scale suppressed. The latter vanish in the gravity-decoupled limit and one is left with a goldstino superfield besides the matter and vector superfields of the model. The gravitino is then very light, in the milli-eV range if SUSY breaking is in the multi-TeV region.

In this work we consider a variation of the minimal supersymmetric standard model (MSSM) called “non-linear MSSM” defined in [6] (see also [7–9]) in which $\sqrt{f}$ is a free parameter that can be as low as few times the scale of soft breaking terms in the visible sector, denoted generically $m_\mathrm{soft}$. We assume that all fields beyond the MSSM spectrum (if any) are heavier than $\sqrt{f}$ (including the sgoldstino). Then, at energies of few TeV, $E\sim m_\mathrm{soft}<\sqrt{f}$ we have the MSSM fields and the (non-linear) goldstino superfield ($X$) coupled to them. The auxiliary component field $F_{X}$ (with $\langle F_X\rangle \sim - f$) of $X$ can mediate interactions (${\propto } 1/f$) between the MSSM fields and generate sizeable effective couplings, in particular in the Higgs sector, if $\sqrt{f}$ is low (few TeV). The study of their implications for the electroweak (EW) fine-tuning is one main purpose of this work. This energy regime can be described by a non-linear goldstino superfield^1^ that satisfies $X^2=0$ [8–11]. This constraint decouples (integrates out) the scalar component of $X$ (sgoldstino), independent of the visible sector details (it depends only on the hidden sector [12–14]). The alternative case of a light sgoldstino, one that can mix with the Standard Model (SM) Higgs, was studied in [7, 15, 16]. At even lower energies, below the sparticle masses one is left with the goldstino fermion coupled to SM fields only, and all supermultiplets are realized non-linearly, i.e. all superpartners are integrated out.

However, with so far negative searches for supersymmetry at the TeV scale, the original motivation for SUSY, of solving the hierarchy problem, is sometimes questioned, since the stability at the quantum level of the hierarchy EW scale $\ll M_P$ becomes more difficult to respect. Indeed, the EW scale $v^2=-m^2/\lambda$, where $m$ is a combination of soft masses ($m_\mathrm{soft}$), therefore $m\sim$ TeV and $\lambda \sim \mathcal{O}(1)$, an effective quartic Higgs coupling; with an increasing $m\sim m_\mathrm{soft}$, it is more difficult to obtain $v=246$ GeV. This tension is quantified by EW scale fine-tuning measures, hereafter denoted generically $\Delta$, with two examples being $\Delta _m$, $\Delta _q$ [17–20] (early studies in [21–25]) defined as

1 $$\begin{aligned} \Delta _m&= \max \big \vert \Delta _{\gamma ^2}\big \vert , \quad \Delta _q=\left\{ \sum _{\gamma } \Delta _{\gamma ^2}^2\right\} ^{1/2},\nonumber \\&\times \mathrm{with}\ \Delta _{\gamma ^2}\equiv \frac{\partial \ln v^2}{\partial \ln \gamma ^2}. \end{aligned}$$

$\Delta _q$ and $\Delta _m$ quantify the variation of $v$ under small relative variations of the ultraviolet (UV) parameters $\gamma$ that denote the SUSY breaking parameters and the (bare) higgsino mass ($\mu _0$). $\Delta _{m,q}$ are regarded as intuitive measures of the success of SUSY as a solution to the hierarchy problem. For the constrained MSSM, $\gamma$ denotes the set: $m_0$, $m_{12}$, $\mu _0$, $A_t$, $B_0$. For the recently measured Standard Model-like Higgs mass $m_h\approx 126$ GeV [26–29], *minimal* values of $\Delta _{m,q}$ in the constrained MSSM are $\approx 800$–$1000$ [30], reduced to ${\approx }500$ for non-universal boundary conditions for gauginos. These values are rather far from those regarded by theorists as more “acceptable” (but still subjective) of $10$ to $100$.

One can ask, however, what relevance such values of the EW fine-tuning have for the realistic character of a model and whether less subjective, model-independent bounds actually exist. Recent results [31–33] (based on previous [30, 34–37]) suggest that there is an interesting link between the EW fine-tuning and the minimal value of chi-square ($\chi ^2_\mathrm{min}$) to fit the EW observables. Under the condition that motivated SUSY of *fixing* the EW scale $v=v(\gamma )$ to its value (246 GeV) and with some simplifying assumptions it was found that there exists a model-independent upper bound $\Delta _q\ll \exp (n_{df})$ [31–33]; here $n_{df}$ is the number of degrees of freedom of the model, $n_{df}=n_\mathcal{O}-n_p$ with $n_\mathcal{O}$ the number of observables and $n_p$ the number of parameters. Generically, $n_{df}\sim 10$ or so; see for example Table 1 in [32], depending on the boundary conditions of the MSSM-like model. This gives $\Delta _q\ll \exp 5\approx 150$ or so. This is an estimate of the magnitude one should seek for $\Delta$ and supports the common view mentioned above that a tuning $\Delta _q\approx 100$ is “acceptable”. It should be noted, however, that the nearly exponential dependence of minimal $\Delta _{m,q}\approx \exp (m_h/\mathrm{GeV})$ noticed in [38–41] and the theoretical error of 2–3 GeV of the Higgs mass [42–44] bring an error factor to the “acceptable” value of $\Delta$ as large as $\exp (2)\approx 7.4$ (or $\exp (3)\approx 20$). Therefore any value of $\Delta$ should be regarded with due care. Nevertheless, the above results tell us that a small $\Delta$ is preferable.

This view is further confirmed by a less conservative approach, which shows that there is also a link between the EW fine-tuning and the covariance matrix of a model [45, 46] in the basis of UV parameters ($\gamma$). This matrix was shown [46] to automatically contain contributions due to the EW fine-tuning w.r.t. parameters $\gamma$ and, in particular, the trace of its inverse contains a contribution proportional to $\Delta _q$. As a result, imposing a fixed, s-standard deviation of the value of chi-square $\chi ^2$ from its minimal value $\chi ^2_\mathrm{min}$, i.e. $\delta \chi ^2\le s^2$ ($\chi ^2=\chi ^2_\mathrm{min}+\delta \chi ^2$), then requires in the loop order considered that $\Delta _q$ have an upper bound [46]. This is a model-independent result and supports our motivation here of seeking models with low $\Delta$.

A very large EW fine-tuning, which increases further with negative searches for SUSY may suggest that we do not understand well the mechanism of SUSY breaking (assuming that SUSY exists not far above the TeV scale). This motivated us to consider the models with low SUSY breaking scale mentioned above and to evaluate their EW fine-tuning for the recently measured Higgs mass. (An early, pre-LHC study of other models with low SUSY scale is found in [47–49].) We examine the values of both $\Delta _m$ and $\Delta _q$ in the “non-linear MSSM” [6] which has a low scale of SUSY breaking, $\sqrt{f}\sim$ few TeV. The only difference of this model from the usual MSSM is present in the gravitino/goldstino and dark matter sectors. We show that this model can have a reduced fine-tuning compared to that in the MSSM-like models. The reduction is done without additional parameters or extra fields in the “visible” sector, which is unlike other models that reduce EW fine-tuning by enlarging the spectrum. Our results depend only on the ratio $m_\mathrm{soft}^2/f$ of the SUSY breaking scale in the visible sector to that in the hidden sector. When $\sqrt{f}$ is low (few TeV) we are in the region of low-scale-SUSY breaking models (with light gravitino) while at large $\sqrt{f}\sim 10^{10}$ GeV we recover the MSSM-like models. We thus have an interpolating parameter between these classes of models. The reason why EW fine-tuning is reduced is the additional quartic Higgs interactions mediated by the auxiliary component of the goldstino superfield, as mentioned earlier; these enhance the *effective* Higgs coupling $\lambda$ and even increase the Higgs mass already at tree level. We stress that this behavior is generic to low-scale SUSY models.

In the next section we review the model. In Sect. 3 we compute analytically the one-loop corrected Higgs mass including $\mathcal{O}(1/f^2)$ corrections from effective operators generated by SUSY breaking. In Sect. 4 we compute at one loop $\Delta _{m,q}$ as functions of the SUSY breaking parameters and $\sqrt{f}$ and then present their numerical values in terms of the one-loop SM-like Higgs mass. For a most conservative case of low $\tan \beta$ and constrained MSSM boundary conditions for the soft terms, we find in “non-linear” MSSM an “acceptable” $\Delta _m\approx 80$ ($\Delta _q\approx 120$) for $\sqrt{f}=2.8$ TeV and $m_h\approx 126$ GeV. This value of $\Delta$ can be reduced further for non-universal gaugino masses and is well below that in the constrained MSSM (for any $\tan \beta$) where $\Delta _{m,q}\sim 800$–$1000$ [30]. This reduction is done without enlarging the MSSM spectrum (for an example with additional massive singlets see [50, 51]).

## The Lagrangian in “non-linear” MSSM

The Lagrangian of the “non-linear MSSM” model can be written as [6–9]

2 $$\begin{aligned} \mathcal{L}=\mathcal{L}_0+\mathcal{L}_X+\mathcal{L}_1+\mathcal{L}_2; \end{aligned}$$

$\mathcal{L}_0$ is the usual MSSM SUSY Lagrangian which we write below to establish the notation:

3 $$\begin{aligned} \mathcal{L}_0&= \sum _{\Phi , H_{1,2}} \int \mathrm{d}^4\theta \Phi ^\dagger e^{V_i}\Phi +\bigg \{\int \mathrm{d}^2\theta \Big [\mu H_1H_2+ H_2QU^c\nonumber \\&+QD^cH_1+LE^cH_1\Big ]+\hbox {h.c.}\bigg \} \nonumber \\&+\sum _{i=1}^3\frac{1}{16g_i^2\kappa } \int \mathrm{d}^2\theta \text{ Tr }[W^\alpha W_\alpha ]_i\nonumber \\&+\hbox { h.c.}, \quad \Phi :Q,D^c,U^c,E^c,L, \end{aligned}$$

$\kappa$ is a constant canceling the trace factor, and the gauge coupling is $g_i$, $i=1,2,3$ for $U(1)_Y$, $SU(2)_L$, and $SU(3)$, respectively. Further, $\mathcal{L}_X$ is the Lagrangian of the goldstino superfield $X=(\phi _X,\psi _X,F_X)$ that breaks SUSY spontaneously and whose Weyl component is “eaten” by the gravitino (super-Higgs effect [52, 53]). $\mathcal{L}_X$ can be written as [8, 9]

4 $$\begin{aligned} \mathcal{L}_X\!=\!\int \mathrm{d}^4\theta X^\dagger X \!+\!\Big \{\int \mathrm{d}^2\theta fX\!+\!\hbox {h.c.}\Big \}\quad \mathrm{with}\ X^2\!=\!0.\quad \end{aligned}$$

**Fig. 1:**
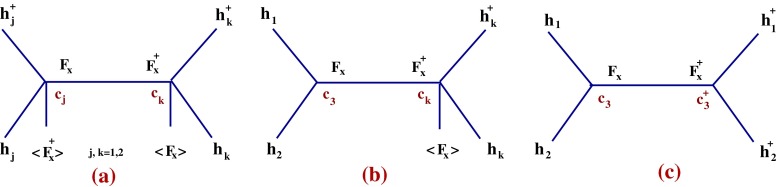
The diagrams that generate the new quartic effective Higgs couplings in V, Eq. (9). The coefficients $c_{1,2,3}$ are generated by $\mathcal{L}_1$, $\mathcal{L}_2$. $F_X$ is the auxiliary component of $X$ that breaks SUSY. The *left* (*right*) diagrams are generated by D (F) terms in the action, while the *middle one* is a mixture of both. These interactions are important in low-scale SUSY breaking models while in the MSSM they are strongly suppressed since $\langle F_X\rangle$ is large)

The otherwise interaction-free $\mathcal{L}_X$ when endowed with a constraint $X^2=0$ [8–11] describes (on-shell) the Akulov–Volkov Lagrangian of the goldstino [54]; see also [55–61], with non-linear SUSY. The constraint has a solution $\phi _X=\psi _X\psi _X/(2F_X)$ that projects (integrates) out the sgoldstino field which becomes massive and is appropriate for a low energy description of SUSY breaking. Further, $\langle F_X\rangle \sim - f$ fixes the SUSY breaking scale ($\sqrt{f}$) and the breaking is transmitted to the visible sector by the couplings of $X$ to the MSSM superfields, to generate the usual SUSY breaking (effective) terms in $\mathcal{L}_1+\mathcal{L}_2$ (see below). These couplings are commonly parametrized (on-shell) in terms of the spurion field $S=m_\mathrm{soft}\theta \theta$ where $m_\mathrm{soft}$ is a generic notation for the soft masses (later denoted $m_{1,2,3}$, $m_{\lambda _i}$); however, this parametrization obscures the dynamics of $X$ (off-shell effects) relevant below that generates additional Feynman diagrams mediated by $F_X$ (Fig. 1). Such effects are not seen in the leading order (in $1/f$) in the spurion formalism. The off-shell couplings are easily recovered by the formal replacement [8, 9]

5 $$\begin{aligned} S\rightarrow \frac{m_\mathrm{soft}}{f} X. \end{aligned}$$

In this way one obtains the SUSY breaking couplings that are indeed identical to those obtained by the equivalence theorem [1–5] from a theory with the corresponding explicit soft breaking terms and in which the goldstino fermion couples to the derivative of the supercurrent of the initial theory. These couplings are generated by the D-terms below:

6 $$\begin{aligned} \mathcal{L}_{1}&= \sum _{i=1,2} c_i \int \mathrm{d}^4\theta X^\dagger X H_i^\dagger e^{V_i}H_i \nonumber \\&+\sum _{\Phi } c_\Phi \int \mathrm{d}^4\theta X^\dagger X\Phi ^\dagger e^V \Phi . \end{aligned}$$

and by the F-terms:

7 $$\begin{aligned} \mathcal{L}_{2}&= \sum _{i=1}^3 \frac{1}{16 g^2_i\kappa } \frac{2m_{\lambda _i}}{f} \int \mathrm{d}^2\theta X\text{ Tr }[W^\alpha W_\alpha ]_i \nonumber \\&+c_3\int \mathrm{d}^2\theta XH_1H_2+\frac{A_u}{f}\int \mathrm{d}^2\theta XH_2QU^c\nonumber \\&+ \frac{A_d}{f}\int \mathrm{d}^2\theta XQD^c H_1 +\frac{A_e}{f}\int \mathrm{d}^2\theta XLE^cH_1+\hbox {h.c.}\nonumber \\ \end{aligned}$$

with

8 $$\begin{aligned}&c_{j}=-\frac{m_j^2}{f^2},\quad j=1,2;\qquad c_3=-\frac{m_3^2}{f}, \qquad c_\Phi =-\frac{m_\Phi ^2}{f^2},\nonumber \\&\qquad \Phi : Q, U^c, D^c, L, E^c, \end{aligned}$$

In the UV one can eventually take $m_\Phi =m_0=m_1=m_2$, $m_{\lambda _i}=m_{12}$ ($i=1,2,3$) for all gaugino masses, $m_3^2=B_0\,m_0\,\mu _0$ ($\mu \equiv \mu _0$ in the UV) and these define the “constrained” version of the “non-linear” MSSM, discussed later. For simplicity, Yukawa matrices are not displayed; to recover them just replace above any pair of fields $\phi _Q \phi _U\rightarrow \phi _Q\gamma _u \phi _U$, $\phi _Q\phi _D\rightarrow \phi _Q\gamma _d \phi _D$, $\phi _L\,\phi _E\rightarrow \phi _L\gamma _e \phi _E$; similar for the fermions and auxiliary fields, with $\gamma _{u,d,e}$ $3\times 3$ matrices.

The total Lagrangian $\mathcal{L}$ defines the model discussed in detail in [6]. The only difference from the ordinary MSSM is in the supersymmetry breaking sector. In the calculation of the on-shell Lagrangian we restrict the calculations up to and including $1/f^2$ terms. This requires solving for $F_\phi$ of matter fields up to and including $1/f^2$ terms and for $F_X$ up to and including $1/f^3$ terms (due to its leading contribution which is $-f$). In this situation, in the final Lagrangian no kinetic mixing is present at the order used.^2^

## The Higgs masses at one loop in “non-linear” MSSM

From the Lagrangian $\mathcal{L}$ one obtains the Higgs scalar potential of the model^3^:

9 $$\begin{aligned} V&= \big (\vert \mu \vert ^2+m_1^2\big )\vert h_1\vert ^2+ \big (\vert \mu \vert ^2 +m_2^2\big ) \vert h_2\vert ^2\nonumber \\&-\big (m_3^2 h_1.h_2+\hbox {h.c.}\big ) +\frac{1}{f^2}\Big \vert m_1^2\vert h_1\vert ^2+m_2^2\vert h_2\vert ^2\nonumber \\&- m_3^2 h_1.h_2\Big \vert ^2 +\frac{g_1^2+g_2^2}{8}\Big [\vert h_1\vert ^2-\vert h_2\vert ^2\Big ]^2+\frac{g_2^2}{2}\vert h_1^\dagger h_2\vert ^2\nonumber \\&+\frac{g_1^2+g_2^2)}{8}\delta \vert h_2\vert ^4 +\mathcal{O}(1/f^3) \end{aligned}$$

with $$h_1.h_2=h_1^0 h_2^0-h_1^- h_2^+$$, $$\vert h_1\vert ^2=\vert h_1^0\vert ^2+\vert h_1^-\vert ^2$$, $$\vert h_2\vert ^2=\vert h_2^0\vert ^2+\vert h_2^+\vert ^2$$.

What is interesting in the above Higgs potential is the presence of the first term in the second line of $V$, absent in MSSM, which is generated by the diagrams in Fig. 1. Therefore, quartic Higgs terms are generated by the dynamics of the goldstino superfield and are not captured by the usual spurion formalism in the MSSM. The impact of these terms for phenomenology is important and analyzed below, for when $\sqrt{f}\sim$ few TeV. When $\sqrt{f}$ is very large which is the case of MSSM-like models, these terms are negligible and thus not included by the spurion formalism. The ignored higher order terms $\mathcal{O}(1/f^3)$ involve non-renormalizable $h_{1,2}^6$ interactions in $V$ and are not considered here.^4^ Finally, the radiatively corrected $m_{1,2,3}$ and $\mu$ in $V$ depend on the scale (hereafter denoted $t$) while the term $\delta \vert h_2\vert ^4$ is generated at one loop by top–stop Yukawa couplings. We thus neglect other Yukawa couplings and our one-loop analysis is valid for low $\tan \beta$; including two-loop leading log effects $\delta$ is

10 $$\begin{aligned} \delta&= \frac{3 h_t^4}{g^2\pi ^2} \left\{ \ln \frac{M_{\tilde{t}}}{m_t}+ \frac{X_t}{4}+\frac{1}{32\pi ^2}(3h_t^2-16g_3^2)\right. \nonumber \\&\times \left. \Big (X_t+2\ln \frac{M_{\tilde{t}}}{m_t}\Big ) \ln \frac{M_{\tilde{t}}}{m_t}\right\} \end{aligned}$$

where

11 $$\begin{aligned} X_t\equiv \frac{2 (A_t m_0-\mu \cot \beta )^2}{M_{\tilde{t}}^2} \Big (1-\frac{(A_t m_0-\mu \cot \beta )^2}{12 M_{\tilde{t}}^2}\Big ). \end{aligned}$$

$M_{\tilde{t}}^2=m_{\tilde{t}_1}\,m_{\tilde{t}_2}$, and $g_3$ is the QCD coupling and $A_t$ is the dimensionless trilinear top coupling.^5^

The minimum conditions of the potential can be written

12 $$\begin{aligned} -v^2=\frac{m^2}{\lambda },\quad 2\lambda \frac{\partial m^2}{\partial \beta }-m^2\frac{\partial \lambda }{\partial \beta } =0, \end{aligned}$$

with the notation^6^:

13 $$\begin{aligned} m^2&\equiv (m_1^2+\mu ^2)\cos ^2\beta +(m_2^2+\mu ^2)\sin ^2 \beta -m_3^2\sin 2\beta ,\nonumber \\ \lambda&\equiv \frac{g_1^2+g_2^2}{8} \Big [\cos ^2 2\beta +\delta \sin ^4 \beta \Big ]\nonumber \\&+\frac{1}{f^2}\Big \vert m_1^2\cos ^2\beta +m_2^2 \sin ^2 \beta -(1/2) m_3^2\sin 2\beta \Big \vert ^2.\nonumber \\ \end{aligned}$$

The correction to the effective quartic Higgs coupling $$\lambda $$, due to the soft terms ($$m_{1,2,3}$$) has implications for the Higgs mass and EW fine-tuning. This positive correction could alleviate the relation between $$v^2$$ and $$m^2$$: indeed, with $$m\sim \mathcal{O}$$(1 TeV) and $$\lambda \sim \mathcal{O}(1)$$, $$v$$ can only be of order $$\mathcal{O}(1$$ TeV$$)$$ as well. This brings about a tension between the EW scale and soft terms ($${\sim }m$$) which cannot easily be separated from each other; this tension is encoded by the EW fine-tuning measures, discussed in Sect. 4. Increasing $$\lambda $$ can alleviate this tension, with impact on the EW fine-tuning. Such a correction to $$\lambda $$ also arises in models with high scale breaking in the hidden sector, so it is present even in usual MSSM but is extremely small in that case since then $$\sqrt{f}\sim 10^{10}$$ GeV. Here we consider $$\sqrt{f}\sim $$ few TeV, which is safely above the current lower bound of $${\approx }700$$ GeV [6, 49, 58, 70].

The two minimum conditions of the scalar potential lead to

14 $$\begin{aligned}&m_1^2-m_2^2= \cot 2\beta \nonumber \\&\quad \times \bigg [-m_3^2+\frac{f^2}{v^2} \frac{(-1+ \sqrt{w}_0)[m_3^2 + m_Z^2\sin 2\beta \big (1-(\delta \sin ^2\beta )/(2\cos 2\beta )\big ) ]}{2\mu ^2+m_Z^2 (\cos ^2 2\beta + \delta \sin ^4\beta )-m_3^2\sin 2\beta }\bigg ],\nonumber \\&m_1^2+m_2^2= \frac{1}{\sin 2\beta }\nonumber \\&\quad \times \bigg [m_3^2 +\frac{f^2}{v^2}\frac{(-1+\sqrt{w}_0)[ -m_3^2 +\big (2\mu ^2 +(\delta /2) m_Z^2 \sin ^2\beta \big )\sin 2\beta ] }{ 2\mu ^2+m_Z^2 (\cos ^2 2\beta +\delta \sin ^4\beta )-m_3^2\sin 2\beta }\bigg ] \end{aligned}$$

where

15 $$\begin{aligned} w_0\!\equiv \!1\!-\!\frac{v^2}{f^2}\big (4\mu ^2\!+\!2m_Z^2(\cos ^2 2\beta \!+\!\delta \sin ^4\beta ) \!-\!2m_3^2\sin 2\beta \!\big ).\nonumber \\ \end{aligned}$$

There is a second solution for $m^2_{1,2}$ at the minimum (with minus in front of $\sqrt{w}_0$) which, however, is not a perturbation of the MSSM solution and is not considered below (since it brings a shift proportional to $f$ of the soft masses, which invalidates the expansion in $m_{1,2}^2/f$).

The mass of the pseudoscalar Higgs is, including a one-loop correction (due to $\delta$):

16 $$\begin{aligned} m_A^2=\frac{2 m_3^2}{\sin 2\beta }\left\{ \frac{3+\sqrt{w}_0}{4}-\frac{m_3^2 v^2}{4 f^2}\sin 2\beta \right\} , \end{aligned}$$

which can be expanded to $\mathcal{O}(1/f^3)$ using the expression of $w_0$. For large $f$ one recovers its MSSM expression at one loop. Further, we computed the masses $m_{h,H}$ including the one-loop correction (due to $\delta$) to find

17 $$\begin{aligned} m_{h,H}^2&\!=\!&\frac{1}{2}\Big [ m_A^2\!+\!m_Z^2\mp \sqrt{w} \!+\! \delta m_Z^2\sin ^2\beta \Big ]\!+\!\Delta m^2_{h,H}\quad \end{aligned}$$

with upper (lower) sign corresponding to $m_h$ ($m_H$) and the correction $\Delta m_{h,H}^2= \mathcal{O}(1/f^2)$ is

18 $$\begin{aligned}&\Delta m^2_{h,H} = \frac{v^2}{64f^2} \Big \{8 \Big [8\mu ^4 -2 m_A^2\mu ^2+4\mu ^2m_Z^2+m_Z^4\nonumber \\&\quad +(2m_A^2\mu ^2+4\mu ^2m_Z^2+m_Z^4)\cos 4\beta \big ]\nonumber \\&\quad -16\delta m_Z^2\big [ m_A^2-4\mu ^2+(m_A^2+2m_Z^2)\cos 2\beta \big ]\sin ^4\beta \nonumber \\&\quad + 16\delta ^2 m_Z^4\sin ^6\beta \!\pm \! (1/\sqrt{w}) \Big [3m_A^6\!-\!m_A^4 (16\mu ^2\!+\!m_Z^2)\nonumber \\&\quad + 4m_A^2 (16\mu ^4+4\mu ^2 m_Z^2+m_Z^4)-8 m_Z^4 (4 \mu ^2 +m_Z^2) \nonumber \\&\quad -4 \big [ m_A^6+m_A^4(m_Z^2-4\mu ^2)-2 m_A^2 m_Z^2(6\mu ^2+m_Z^2)\nonumber \\&\quad + 2 m_Z^2 (8\mu ^4+4\mu ^2 m_Z^2+m_Z^4)\big ]\cos 4\beta \nonumber \\&\quad +m_A^2 (m_A^2+m_Z^2)(m_A^2+4m_Z^2)\cos 8\beta \nonumber \\&\quad +4\delta m_Z^2\big [-m_A^4 - 2 m_Z^4 +m_A^2 (8\mu ^2+m_Z^2)\nonumber \\&\quad +\big ( (m_A^2-4\mu ^2)^2 -3 (m_A^2-8\mu ^2) m_Z^2+7m_Z^4\big )\cos 2\beta \nonumber \\&\quad +\big (m_A^4+(3m_A^2-8\mu ^2)m_Z^2 \nonumber \\&\quad -2 m_Z^4\big )\cos 4\beta - (m_A^4+m_A^2 m_Z^2 -m_Z^4)\cos 6\beta \big ]\sin ^2\beta \nonumber \\&\quad +16\delta ^2 m_Z^4 (m_A^2-4\mu ^2+3m_Z^2 \cos 2\beta )\sin ^6\beta \nonumber \\&\quad -16\delta ^3 m_Z^6\sin ^8\beta \Big ]\Big \}+\mathcal{O}(1/f^3), \end{aligned}$$

with

19 $$\begin{aligned} w&\equiv (m_A^2+m_Z^2)^2-4 m_A^2m_Z^2\cos ^2 2\beta +2\delta (m_A^2-m_Z^2)\nonumber \\&\times m_Z^2\cos (2\beta )\sin ^2\beta +\delta ^2 m_Z^4\sin ^4\beta . \end{aligned}$$

It is illustrative to take the limit of large $\tan \beta$ on $m_{h,H}^2$ with $m_A$ fixed. One finds

20 $$\begin{aligned} m_{h}^2&= \Big [(1+\delta ) m_Z^2 + \frac{v^2}{2 f^2} \big (2 \mu ^2+ (1+\delta ) m_Z^2\big )^2\nonumber \\&+\mathcal{O}(\cot ^2\beta )\Big ] +\mathcal{O}(1/f^3), \nonumber \\ m_H^2&= \big [m_A^2+\mathcal{O}(\cot ^2\beta )\big ]+\mathcal{O}(1/f^3), \end{aligned}$$

where we ignored the $\tan \beta$ dependence of $\delta$. Due to the $\mathcal{O}(\cot ^2\beta )$ suppression, Eq. (20) is valid even at smaller $\tan \beta \sim 10$. In this limit a significant increase of $m_h$ to $120$ or even $126$ GeV is easily achieved, driven by classical effects alone with $\mu$ near TeV (and eventually small quantum corrections, $\delta \sim 0.5$). Such an increase due to $\mu$ is thus of SUSY origin, even though the quartic Higgs couplings ($\mathcal{O}(1/f^2)$) giving this effect involved the soft masses $m_{1,2,3}$. These combined to give, at the EW minimum, the $\mu$-dependent increase in Eq. (20). For large $f$ one recovers the MSSM value of $m_{h,H}$, at one loop. Equations (17) and (18) are used in Sect. 4 to analyze the EW fine-tuning as a function of $m_h$.

## The electroweak scale fine-tuning

### General results

To compute the EW fine-tuning we use two definitions for it already shown in Introduction:

21 $$\begin{aligned} \Delta _m&= \max \big \vert \Delta _{\gamma ^2}\big \vert , \quad \Delta _q=\left\{ \sum _{\gamma } \Delta _{\gamma ^2}^2\right\} ^{1/2},\nonumber \\&\mathrm{with}\ \Delta _{\gamma ^2}\equiv \frac{\partial \ln v^2}{\partial \ln \gamma ^2}, \end{aligned}$$

where $\gamma = m_0, m_{12}, A_t, B_0, \mu _0$ for the constrained “non-linear” MSSM. In the following we evaluate $\Delta _m$, $\Delta _q$ at the one-loop level in our model. Using Eq. (12), which give $m^2=m^2(\gamma , \beta )$ and $\lambda =\lambda (\gamma ,\beta )$, one has a general result for $\Delta _{\gamma ^2}$ which takes into account that $\tan \beta$ depends on $\gamma$ via the second min condition in Eq. (12). The result is [47, 48]

22 $$\begin{aligned}&\Delta _{\gamma ^2}=-\frac{\gamma }{2z}\bigg [\bigg (2\frac{\partial ^{2}m^{2}}{\partial \beta ^{2}}+v^{2}\frac{\partial ^{2}\lambda }{\partial \beta ^{2}}\bigg ) \bigg (\frac{\partial \lambda }{\partial \gamma }+\frac{1}{v^{2}}\frac{\partial m^{2}}{\partial \gamma }\bigg )\nonumber \\&\quad +\frac{\partial m^{2}}{\partial \beta }\frac{ \partial ^{2}\lambda }{\partial \beta \partial \gamma }-\frac{\partial \lambda }{ \partial \beta }\frac{\partial ^{2}m^{2}}{\partial \beta \partial \gamma }\bigg ] \end{aligned}$$

where

23 $$\begin{aligned} z\equiv \lambda \bigg (2\frac{\partial ^{2}m^{2}}{\partial \beta ^{2}}+v^{2}\frac{\partial ^{2}\lambda }{\partial \beta ^{2}}\bigg )-\frac{v^{2}}{2}\bigg (\frac{\partial \lambda }{\partial \beta }\bigg )^{2}. \end{aligned}$$

Using these expressions, one obtains $\Delta _m$ and $\Delta _q$.

Let us first consider the limit of large $\tan \beta$, so the first relation in Eq. (12) becomes

24 $$\begin{aligned} v^2=-\frac{2(m_2^2+\mu ^2)}{(1+\delta )(g_1^2+g_2^2)/4+2m_2^4/f^2}+\mathcal{O}(\cot \beta ), \end{aligned}$$

which gives

25 $$\begin{aligned} \Delta _{\gamma ^2}\!=\!-\!\frac{\partial (m_2^2+\mu ^2)}{\partial \ln \gamma } \frac{(1\!+\!2v^2m_2^2/f^2)^s}{(1\!+\!\delta )m_Z^2\!+\!2v^2m_2^4/f^2} +\mathcal{O}(\cot \beta ),\nonumber \\ \end{aligned}$$

where $s=1\ \hbox {if}\ \gamma \not =\mu _0;\,\, s=0\ \hbox {if}\ \gamma =\mu _0$, and $\mu$, $m_2$ are functions of the scale.^7^ If also $f$ is large, one recovers the MSSM corresponding expression (ignoring a $\tan \beta$ dependence of $\delta$):

26 $$\begin{aligned} \Delta ^0_{\gamma ^2}=-\frac{\partial (m_2^2+\mu ^2)}{\partial \ln \gamma }\frac{1}{(1+\delta )m_Z^2} +\mathcal{O}(\cot \beta ), \end{aligned}$$

which is interesting on its own. For the EW symmetry breaking to exist one must have $m_2^2+\mu ^2<0$ and therefore $\Delta _{\gamma ^2}$ of the “non-linear MSSM” is smaller than in the MSSM with similar UV boundary conditions for parameters $\gamma$. Indeed, in this case the ratio $r$ of $\Delta _{\gamma ^2}$ to that in a MSSM-like model denoted $\Delta _{\gamma ^2}^0$,

27 $$\begin{aligned} r=\frac{\Delta _{\gamma ^2}}{\Delta _{\gamma ^2}^0}= \frac{(1+2 v^2 m_2^2/f^2)^s(1+\delta )m_Z^2}{(1+\delta )m_Z^2+2v^2m_2^4/f^2} +\mathcal{O}(\cot \beta ),\nonumber \\ \end{aligned}$$

is smaller than unity: $r\approx 1/2$ if $\delta \approx 0.8$, $\vert m_2^2\vert /f\approx 0.35$, and $r\approx 1/3$ if $\delta \approx 0.8$, $\vert m^2_2\vert /f\approx 0.5$ with $\sqrt{f}$ above the TeV scale (recall $\vert m_2^2\vert /f<1$ for convergence and $\delta \sim 0.5$–$1$). So for a large $\tan \beta$ the EW fine-tuning associated to each UV parameter is smaller relative to the MSSM and the same can then be said about overall $\Delta _m$ and $\Delta _q$. This reduction is actually more significant, since for the same point in the parameter space the Higgs mass is larger in the “non-linear” MSSM than in the MSSM alone, already at the tree level. Indeed, we saw in Eq. (20) that even in the absence of loop corrections one can easily achieve $m_h\approx 120$ GeV, without the additional, significant fine-tuning “cost”, present for $m_h>115$ GeV in the MSSM. This “cost” is $\Delta \sim \exp (\delta m_h/\mathrm{GeV})$ due to loop corrections needed to increase $m_h$ by $\delta m_g$ in MSSM models;^8^ for the same $m_h$ the reduction is then expected to be by a factor $\Delta \sim \exp (120$–$115)\sim 150$ relative to the constrained MSSM case. Then our $\Delta _{m,q}$ can be smaller by this factor and $r$ is also much smaller than unity when evaluated for the same $m_h$. Finally, fixing $m_h$ to its measured value is a very strong constraint on the parameter space, which, once satisfied, allows other EW constraints to be automatically respected [30], so this conclusion is unlikely to be affected by them.

Let us mention that in MSSM-like models the EW fine-tuning $\Delta$ is usually reduced as one increases $\tan \beta$ for a fixed $m_h$ (all the other parameters allowed to vary) [38–41]. This is because at large $\tan \beta$ additional Yukawa couplings effects (down sector) are enhanced and help the radiative EW symmetry breaking (thus reducing $\Delta$), while at small $\tan \beta$ this effect is suppressed [30]. The situation is similar to the above “non-linear” MSSM model.^9^

### The constrained “non-linear” MSSM

The reduction of the EW fine-tuning in our model can be illustrated further by comparing it with that in the constrained MSSM (CMSSM) with universal UV scalar mass $m_0$ and gaugino mass $m_{12}$ and including only the top–stop Yukawa coupling correction. In that case one has

28 $$\begin{aligned} m_1^2(t)&= m_0^2 +m_{12}^2\sigma _1(t),\quad \mu ^2(t)=\mu _0^2\sigma _8^2(t),\nonumber \\ m_2^2(t)&= m_{12}^2\sigma _4(t)\!+\!A_tm_0m_{12}\sigma _5(t) \!+\!m_0^2\sigma _7(t)\! -\! m_0^2A_t^2\sigma _6(t),\nonumber \\ m_3^2(t)&= \mu _0m_{12}\sigma _2(t)+B_0m_0\mu _0\sigma _8(t)+ \mu _0m_0A_t\sigma _3(t) \end{aligned}$$

where we made explicit the dependence of the soft masses $m_{1,2,3}$ and $\mu$ and of the coefficients $\sigma _i$ on the momentum scale $t=\ln \Lambda _{UV}^2/q^2$ induced by radiative corrections; $\sigma _i$ also depend on $\tan \beta$ and so do the soft masses. The high scale boundary conditions are chosen such as $\sigma _{1,2,3,4,5,6}(0)=0$, $\sigma _{7,8}(0)=1$ when quantum corrections are turned off. For $q^2=m_Z^2$ the values of $\sigma _i$ are given in the Appendix. These expressions are used in our numerical analysis below.

#### The large $\tan \beta$ case

This regime was already discussed in the general case in Sect. 4.1. A numerical analysis of this case involves additional Yukawa couplings of the “down” sector not included in our $V$ and is beyond the goal of this paper. However, we can still provide further insight for the constrained “non-linear MSSM”. From Eq. (25), one has

29 $$\begin{aligned} \Delta _{\mu _0^2}&= -\frac{2\mu _0^2\sigma _8^2}{(1+\delta )m_Z^2 +2v^2 m_2^4/f^2}+\mathcal{O}(\cot ^2\beta )\nonumber \\ \Delta _{m_0^2}&= -\frac{m_0(1+2 v^2 m_2^2/f^2)}{(1+\delta )m_Z^2+2v^2m_2^4/f^2}\nonumber \\&\times (A_t\sigma _5-2 A_t^2\,m_0\sigma _6+2 m_0\sigma _7)+\mathcal{O}(\cot \beta ) \nonumber \\ \Delta _{m_{12}^2}&= -\frac{m_{12}\,(1+2 v^2 m_2^2/f^2)}{(1+\delta )m_Z^2+2v^2m_2^4/f^2}\nonumber \\&\times (2 m_{12}\sigma _4 +A_tm_0 \sigma _5)+\mathcal{O}(\cot \beta ) \nonumber \\ \Delta _{A_t^2}&= -\frac{A_t(1+2 v^2 m_2^2/f^2)}{(1+\delta )m_Z^2+2v^2m_2^4/f^2}\nonumber \\&\times (m_{12}\sigma _5-2 m_0A_t\sigma _6)m_0+\mathcal{O}(\cot \beta ),\nonumber \\ \Delta _{B_0^2}&= \mathcal{O}(\cot \beta ); \end{aligned}$$

$m_2^2$ is given in Eq. (28) and, since $m_2^2<0$, the absolute values of the above $\Delta$’s and then of $\Delta _{m,q}$ are smaller than those in the limit $f\rightarrow \infty$ when one recovers the constrained MSSM model (at large $\tan \beta$). So fine-tuning is reduced as already argued in the general discussion.

**Fig. 2:**
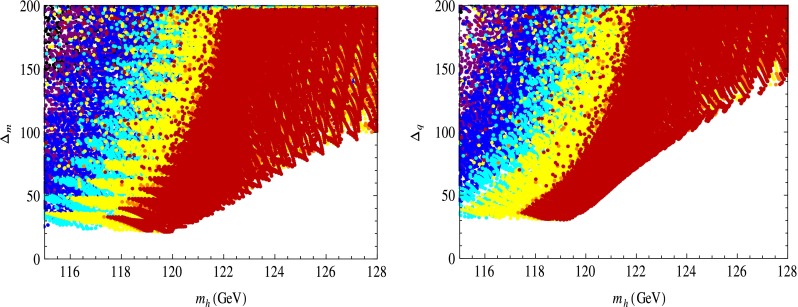
The EW fine-tuning $\Delta _m$ (*left*) and $\Delta _{q}$ (*right*) as functions of the SM-like Higgs mass $m_h$ (in GeV), all evaluated at one loop, for $\tan \beta \le 10$. These plots have a fixed value $\sqrt{f}=2.8$ TeV of the SUSY breaking scale and $\tan \beta$ increases from *left* ($\tan \beta \le 2.5$) to right ($\tan \beta =10$) as shown by *different colors*: *black*/leftmost region: $\tan \beta \le 2.5$; *purple*: $2.5\le \tan \beta \le 4$; *blue*: $4\le \tan \beta \le 4.5$; *cyan*: $4.5\le \tan \beta \le 5.5$; *yellow*: $5.5\le \tan \beta \le 9.5$; *red*/rightmost region: $\tan \beta =10$ (a larger $\tan \beta$ region is on top of that of smaller $\tan \beta$). For $m_h=126$ GeV, minimal $\Delta _m\approx 80$ and $\Delta _q\approx 120$, while in the corresponding constrained MSSM minimal values (for $\tan \beta <55$), $\Delta _m\sim \Delta _q\approx 800$–$1000$, too large to be shown here; for details see figures 1–8 in [30]. The wide range of values for $m_h$ was chosen only to display the $\tan \beta$ dependence and to allow for the 2–3 GeV theoretical error of $m_h$ [42–44]

Turning off the quantum corrections to soft masses and $\mu$ ($\sigma _{1,2,\ldots ,6}=0$, $\sigma _{7,8}=1$) and quartic coupling ($\delta =0$), for large $f$, the above relations simplify to give for constrained MSSM

30 $$\begin{aligned} \vert \Delta _{\gamma ^2}\vert =\frac{2 \gamma ^2}{m_Z^2}+\mathcal{O}(\cot \beta ),\quad \gamma =m_0, \mu _0 \end{aligned}$$

with the remaining expressions being $\mathcal{O}(\cot \beta )$. This also shows that in the constrained MSSM, the dominant contributions to fine-tuning (at classical level) are due to $m_0$ and $\mu _0$. In general, $\Delta _{m_0^2}$ is related to QCD effects that increase fine-tuning and dominates for $m_h>115$ GeV (fig.2 in the first reference in [38–41]). For TeV-valued $m_0=\mu _0=2$ TeV ($\delta =0$) one then has $\Delta _{q}=683$, which gives a good estimate of the value of fine-tuning in constrained MSSM.^10^ Equation (30) has close similarities to other fine-tuning measures defined in the literature such as $\Delta _{EW}$ of [71–73].

#### The small $\tan \beta$ case

From Eqs. (21), (22), and (23) we find the following analytical results for $\Delta _{\gamma ^2}$ at one-loop level:

31 $$\begin{aligned}&\Delta _{\mu _0^2}=-\frac{4}{Dv^2} \Big \{-2 f^2 y_1 \sin 2\beta \Big [ (4+\delta ) f^2 m_Z^2 \nonumber \\&\quad + 2 v^2 (y_1^2+ y_2^2)-2 (\delta f^2 m_Z^2 +v^2y_2y_3) \cos 2\beta \nonumber \\&\quad +\big [ (4+\delta ) f^2 m_Z^2+2 v^2 y_1^2 \big ]\cos 4\beta -2 v^2 y_1 y_2 \sin 4\beta \Big ]\nonumber \\&\quad + \Big [ \big [ f^2 (m_Z^2\delta +4 y_2)+2 v^2 y_2 y_3\big ] \cos 2\beta \nonumber \\&\quad -\big [ (4+\delta ) f^2 m_Z^2 +2 v^2 (-y_1^2+ y_2^2)\big ]\cos 4\beta \nonumber \\&\quad + 2 y_1 (4 f^2+v^2 y_3 -4 v^2 y_2 \cos 2\beta ) \sin 2 \beta \Big ]\nonumber \\&\quad \times \Big [ 8 f^2 \mu _0^2 {\sigma }_8^2 +v^2 y_1^2 + y_1 \big [- 4 f^2 \sin 2\beta \nonumber \\&\quad + v^2 (-y_1 \cos 4\beta -2 y_3\sin 2\beta +y_2 \sin 4\beta )\big ]\Big ]\Big \},\end{aligned}$$

32 $$\begin{aligned}&\Delta _{m_0^2}= - \frac{4 f^2 m_0}{D} \Big \{ 4 \Big [ \big [ f^2 (m_Z^2\delta +4 y_2)+2 v^2 y_2 y_3\big ]\cos 2\beta \nonumber \\&\quad - \big [ (4+\delta ) f^2 m_Z^2+2 v^2 (y_2^2-y_1^2)\big ] \cos 4\beta \nonumber \\&\quad +2 y_1 \big [4 f^2+v^2 (y_3-4 y_2 \cos 2\beta ) \big ]\sin 2\beta \Big ]\nonumber \\&\quad \times \Big [ v^{-2} \big [ 2 m_0 \cos ^2\beta +y_4 \sin ^2\beta \nonumber \\&\quad -\mu _0 (A_t \sigma _3+B_0\sigma _8) \sin 2\beta \big ] \nonumber \\&\quad +(1/f^2) \big [ 2 m_0 \cos ^2\beta -\mu _0 (A_t \sigma _3 +B_0\sigma _8)\nonumber \\&\quad \times \cos \beta \sin \beta +y_4 \sin ^2\beta \big ]\nonumber \\&\quad \times (y_3-y_2 \cos 2\beta -y_1 \sin 2\beta ) \Big ] \nonumber \\&\quad +8(- 2 y_1 \cos 2\beta + y_2 \sin 2\beta ) \Big [ (1/2) \big [\mu _0 (A_t \sigma _3 \nonumber \\&\quad + B_0\sigma _8 ) \cos 2\beta + (2 m_0 -y_4) \sin 2\beta \big ] \nonumber \\&\quad \times (y_2 \cos 2\beta -y_3+y_1\sin 2\beta )-\big [ 2 m_0 \cos ^2\beta \nonumber \\&\quad - \mu _0 (A_t \sigma _3+B_0 \sigma _8 )(1/2) \sin 2\beta + y_4 \sin ^2\beta \big ] \nonumber \\&\quad \times (y_1 \cos 2\beta -y_2 \sin 2 \beta ) \Big ] + (1/v^2) \big [ 2\mu _0 (A_t \sigma _3 \nonumber \\&\quad + B_0 \sigma _8)\cos 2\beta + (2m_0-y_4) \sin 2\beta \big ] \nonumber \\&\quad \times \big [ -2 f^2 m_Z^2 (-\delta +(4+\delta )\cos 2\beta )\sin 2\beta \nonumber \\&\quad +4 v^2 (-y_3 +y_2 \cos 2\beta + y_1\sin 2\beta )(y_1\cos 2\beta \nonumber \\&\quad -y_2 \sin 2\beta ) \Big ] \Big \}, \end{aligned}$$

and

33 $$\begin{aligned}&\Delta _{m_{12}^2}\!=\! \frac{-4 f^2 m_{12}}{D} \Big \{ 4 \Big [ \big [f^2 (m_Z^2\delta \!+\!4 y_2)\!+\! 2 v^2 y_2 y_3\big ]\cos 2\beta \nonumber \\&\quad - \big [ (4+\delta ) f^2 m_Z^2 + 2 v^2 (y_2^2-y_1^2) \big ] \nonumber \\&\quad \times \cos 4\beta +2 y_1 ( 4 f^2 +v^2 y_3 -4 v^2 y_2 \cos 2\beta ) \sin 2\beta \Big ]\nonumber \\&\quad \times \Big [ \frac{1}{v^2} \big [ 2 m_{12} \sigma _1 \cos ^2\beta - \mu _0 \sigma _2 \sin 2\beta \nonumber \\&\quad +(2 m_{12} \sigma _4 + A_t m_0 \sigma _5 )\sin ^2\beta \big ] + (1/f^2) \nonumber \\&\quad \times \big [ 2 m_{12} \sigma _1 \cos ^2\beta - (1/2)\mu _0 \sigma _2 \sin 2\beta \nonumber \\&\quad +(2 m_{12} \sigma _4\! + \!A_t m_0 \sigma _5 )\sin ^2\beta \big ] (y_3\!-\!y_2 \cos 2\beta \!- \!y_1 \sin 2\beta ) \!\Big ]\nonumber \\&\quad + 8 (y_2\sin 2\beta -2 y_1 \cos 2\beta ) \nonumber \\&\quad \times \Big [ (1/2) \big [\mu _0 \sigma _2 \cos 2\beta \!+\! \big (2 m_{12} (\sigma _1\!-\!\sigma _4)\!-\!A_t m_0 \sigma _5 \big )\sin 2\beta \big ]\nonumber \\&\quad \times (-y_3+y_2 \cos 2\beta +y_1 \sin 2\beta ) \nonumber \\&\quad -\Big [ 2 m_{12} \sigma _1 \cos ^2\beta -\frac{1}{2} \mu _0 \sigma _2 \sin 2\beta \nonumber \\&\quad +(2 m_{12}\sigma _4+A_t m_0 \sigma _5)\sin ^2\beta \Big ](y_1 \cos 2\beta -y_2 \sin 2\beta ) \Big ]\nonumber \\&\quad +(1/v^2)\Big [2\mu _0 \sigma _2 \cos 2\beta \!+\!\big [ 2 m_{12} (\sigma _1\!-\!\sigma _4) \!-\!A_t m_0 \sigma _5 \big ] \sin 2\beta \Big ]\nonumber \\&\quad \times \Big [ -2 f^2 m_Z^2 (-\delta +(4+\delta )\cos 2\beta )\sin 2\beta \nonumber \\&\quad + 4 v^2 (-y_3 +y_2\cos 2\beta +y_1 \sin 2\beta )\nonumber \\&\quad \times (y_1 \cos 2\beta -y_2 \sin 2\beta ) \Big ] \Big \}, \end{aligned}$$

and

34 $$\begin{aligned}&\Delta _{A_t^2}=\frac{-4 A_t}{ D} \Big \{ 8f^2 (y_2\sin 2\beta -2y_1\cos 2\beta )\nonumber \\&\quad \times \Big [ (m_0/2)\big (\mu _0\sigma _3\cos 2\beta +(2A_tm_0\sigma _6 - m_{12}\sigma _5) \sin 2\beta \big ) \nonumber \\&\quad \times (-y_3+y_2\cos 2\beta +y_1\sin 2\beta ) +m_0\sin \beta \nonumber \\&\quad \times \big [\mu _0\sigma _3\cos \beta + (-m_{12}\sigma _5+2A_t m_0\sigma _6)\sin \beta \big ] \nonumber \\&\quad \times (y_1\cos 2\beta -y_2\sin 2\beta )\Big ]+(f^2/v^2) m_0\big [2\mu _0\sigma _3\cos 2\beta \nonumber \\&\quad + (-m_{12}\sigma _5+2A_tm_0\sigma _6) \sin 2\beta \big ] \nonumber \\&\quad \times \Big [-2f^2 m_Z^2 \big [-\delta +(4+\delta )\cos 2\beta \big ]\sin 2\beta \nonumber \\&\quad +4v^2(-y_3+y_2\cos 2\beta +y_1\sin 2\beta ) \nonumber \\&\quad \times (y_1\cos 2\beta -y_2\sin 2\beta )\Big ]- (4/v^2)m_0\sin \beta \nonumber \\&\quad \times \Big [\big (f^2(\delta m_Z^2+4y_2)+2v^2y_2y_3\big )\cos 2\beta \nonumber \\&\quad -\big [f^2m_Z^2(4+\delta )+2v^2(-y_1^2+y_2^2)\big ]\cos 4\beta \nonumber \\&\quad +2y_1(4f^2+v^2y_3-4v^2y_2\cos 2\beta )\sin 2\beta \Big ] \nonumber \\&\quad \times \Big [\mu _0\sigma _3\cos \beta \big [ 2 f^2+v^2y_3-v^2 (y_2\cos 2\beta +y_1\sin 2\beta )\big ]\nonumber \\&\quad +(m_{12}\sigma _5-2A_tm_0\sigma _6)\sin \beta \nonumber \\&\quad \times \big [-f^2-v^2y_3 +v^2(y_2\cos 2\beta +y_1\sin 2\beta )\big ]\Big ]\Big \}. \end{aligned}$$

Finally

35 $$\begin{aligned}&\Delta _{B_0^2}=-\frac{8B_0 m_0 \mu _0\sigma _8}{D} \Big \{\frac{\sin 2\beta }{v^2}\Big [\big (f^2(\delta m_Z^2+4y_2)\nonumber \\&\quad +2v^2 y_2 y_3 \big )\cos 2\beta -\big [(4+\delta )f^2m_Z^2 \nonumber \\&\quad +2v^2(-y_1^2+y_2^2)\big ]\cos 4\beta + 2y_1 ( 4f^2+v^2 y_3\nonumber \\&\quad -4v^2y_2\cos 2\beta )\sin 2\beta \Big ] \big [-2f^2-v^2y_3 \nonumber \\&\quad +v^2(y_2\cos 2\beta +y_1 \sin 2\beta )\big ]+\frac{f^2}{v^2} \cos 2\beta \nonumber \\&\quad \times \Big [-2f^2m_Z^2\big [-\delta +(4+\delta )\cos 2\beta \big ]\sin 2\beta +4v^2\nonumber \\&\quad \times (-y_3\!+\!y_2\cos 2\beta \!+\!y_1\sin 2\beta )(y_1\cos 2\beta \!-\!y_2\sin 2\beta )\!\Big ] \nonumber \\&\quad -2f^2(2y_1\cos 2\beta -y_2\sin 2\beta )\nonumber \\&\quad \times (-y_3\cos 2\beta +y_2\cos 4\beta +y_1\sin 4\beta )\Big \}. \end{aligned}$$

The denominator $D$ used in the above formulas is

36 $$\begin{aligned} D&\equiv 2 f^2 \Big [ \big [ f^2 (m_Z^2\delta +4 y_2) +2 v^2 y_2 y_3 \big ] \cos 2\beta \nonumber \\&-\big [ (4+\delta ) f^2 m_Z^2 +2 v^2 (y_2^2-y_1^2)\big ]\cos 4\beta \nonumber \\&+2 y_1 (4 f^2 +v^2 y_3 -4 v^2 y_2 \cos 2\beta )\sin 2\beta \Big ] \nonumber \\&\times \Big [ 8 (m_Z^2/v^2) \big ( \cos ^2 2\beta +\delta \sin ^4\beta \big ) + (4/f^2) (-y_3 \nonumber \\&+y_2\cos 2\beta + y_1\sin 2\beta )^2 \Big ] - (1/v^2) \Big [ -4 v^2 (-y_3 \nonumber \\&+ y_2 \cos 2\beta +y_1 \sin 2\beta )(y_1 \cos 2\beta \nonumber \\&-y_2 \sin 2\beta ) \!+ \!f^2 m_Z^2 \big (-2 \delta \sin 2\beta \!+\!(4+\delta )\sin 4\beta \big )\Big ]^2\!.\nonumber \\ \end{aligned}$$

In the above expressions we introduced the notations:

37 $$\begin{aligned} y_1&\equiv \mu _0 (m_{12} \sigma _2 + A_t m_0 \sigma _3 + B_0 m_0 \sigma _8),\nonumber \\ y_2&\equiv \!-\! m_{12}^2 (\sigma _1\!-\!\sigma _4) \!-\! m_0(m_0 \!-\! A_t m_{12} \sigma _5 \!+\! A_t^2 m_0 \sigma _6 \!-\!m_0 \sigma _7), \nonumber \\ y_3&\equiv y_2 \!+\! 2 \sigma _1 m_{12}^2 \!+\! 2 m_0^2,\nonumber \\ y_4&\equiv A_t m_{12} \sigma _5\! -\! 2 A_t^2 m_0 \sigma _6 \!+\!2 m_0 \sigma _7. \end{aligned}$$

The expressions for $\Delta _{\gamma ^2}$ simplify considerably if one turns off the quantum corrections to the soft terms ($\sigma _{1,2,\ldots ,6}=0$, $\sigma _{7,8}=1$). We checked that in the limit of large $f$, $\Delta _{\gamma ^2}$ recover the analytical results for fine-tuning at one loop found in [62] for the constrained MSSM (plus corrections $\mathcal{O}(1/f^2)$). One also recovers from the above expressions for $\Delta _{\gamma ^2}$ the results in Eq. (29).

### Numerical results

**Fig. 3:**
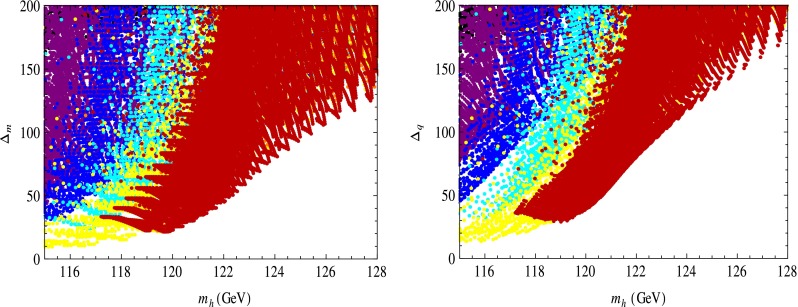
$\Delta _m$ (*left*) and $\Delta _q$ (*right*), with similar considerations as for Fig. 2 but with $\sqrt{f}=3.2$ TeV. In this case, minimal $\Delta _m=105$ and $\Delta _q=145$ for $m_h=126$ GeV

**Fig. 4:**
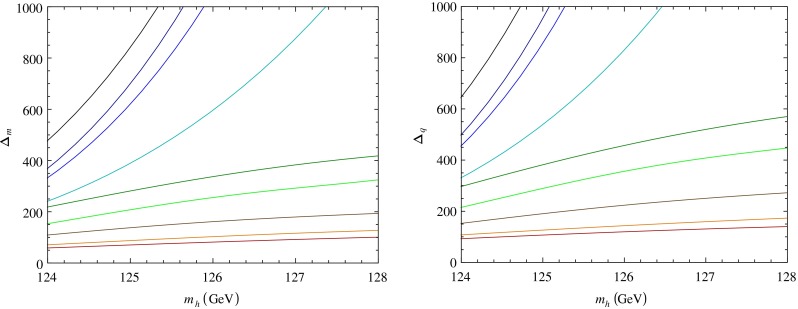
The dependence of *minimal* $\Delta _m$ (*left*) and $\Delta _q$ (*right*) on $m_h$ (GeV) for different $\sqrt{f}$, for fixed $\tan \beta =10$ with the other parameters allowed to vary. We allowed a ${\pm }2$ GeV (theoretical) error for $m_h$ [42–44] about the central value of $126$ GeV. For a fixed $m_h$ the minimal values of $\Delta _m$, $\Delta _q$ increase as we increase $\sqrt{f}$ from the lowest to the top curve, in this order: $2.8$ TeV (the *lower*/*red curve*), $3.2$ TeV (*orange*), $3.9$ TeV (*brown*), $5$ TeV (*green*), $5.5$ TeV (*dark green*), $6.3$ TeV (*cyan*), $7.4$ TeV (*blue*), $8$ TeV (*dark blue*), $8.7$ TeV (*black*/*top curve*). The lowest two curves (*red*, *orange*) correspond to the minimal values of $\Delta _m$ and $\Delta _q$ in Figs. 2 and 3. For large enough $\sqrt{f}\ge 10$ TeV, one recovers the MSSM-like values of $\Delta _m$, $\Delta _q$ for a similar $m_h$

Using the results in Eqs. (31) to (37) we evaluated $\Delta _m$ and $\Delta _q$ for fixed values of the SUSY breaking scale in the hidden sector $\sqrt{f}$ for $\tan \beta \le 10$, subject to the EW constraints (for a discussion of these, see [30]). Note that imposing the Higgs mass range of $126\pm (2\ \hbox {to}\ 3)$ GeV (to allow for the theoretical error [42–44]) automatically respects these constraints [30]. For a rapid convergence of the perturbative expansion in $1/f$ of the Lagrangian we demanded that $m_\mathrm{soft}^2/f<1/4$, where $m_\mathrm{soft}$ stands for SUSY breaking terms.^11^ The results are shown in Figs. 2, 3, and 4.

For $m_h=126$ GeV we find *minimal* values of $\Delta _m\approx 80$ and $\Delta _q\approx 120$ for $\sqrt{f}=2.8$ TeV (Fig. 2) and $\Delta _m\approx 105$ and $\Delta _q\approx 145$ for $\sqrt{f}=3.2$ TeV (Fig. 3). These values of $\sqrt{f}$ are well above the current lower bound of ${\approx }700$ GeV [6, 49, 58, 70]. As one increases $\tan \beta$ for a given $m_h$, $\Delta _m$ or $\Delta _q$ decreases, as shown by the color encoding corresponding to fixed $\tan \beta$ in Figs. 2 and 3; this is also valid in the MSSM as seen in Figures 3, 4, 5 in the first reference in [38–41]. These values for fine-tuning are already “acceptable” and significantly below the *minimal* values in the constrained MSSM where for $m_h\approx 126$ GeV, $\Delta _{m,q}\approx 800$–$1000$, see Figures 1–8 in [30], obtained after scanning over all $2\le \tan \beta \le 55$.

The reduced values of $\Delta _m$ and $\Delta _q$ are due to the fact that $m_h$ is significantly above that of the constrained MSSM already at the classical level, see Eqs. (17) to (20) for $\delta =0$, where values of $120$–$126$ GeV are easily achieved, so only very small quantum corrections are actually needed (unlike in the MSSM). This is a consequence of the (classically) increased effective quartic Higgs coupling. Also notice that minimal values of $\Delta _m$ and $\Delta _q$ have a similar dependence on $m_h$ and are only mildly different in size, as also noticed for the MSSM [30].

In Fig. 4 we presented the minimal values of $\Delta _m$ and $\Delta _q$ as functions of $m_h$ for fixed $\tan \beta =10$ for different values of the SUSY breaking scale from $\sqrt{f}=2.8$ TeV to $8.7$ TeV. When increasing $\sqrt{f}$ to larger values, in the region above $10$ TeV, the effects of the additional quartic terms in the scalar Higgs potential are rapidly suppressed and one recovers the usual constrained MSSM-like scenario with similar UV boundary conditions, with larger fine-tuning for the same $m_h$ and with minimal $\Delta _{q,m}\sim \exp (m_h/\mathrm{GeV})$ (see the top curves in Fig. 4). This exponential behavior is characteristic for MSSM-like models due to (large) quantum corrections to the Higgs mass [38–41]. Relaxing the UV universality boundary condition for the gaugino masses reduces $\Delta _{m,q}$ further, similar to the MSSM [23, 30, 74, 75], by a factor of ${\approx }2$ from the values given by the curves in Fig. 4. Thus, values of $\sqrt{f}$ of up to 5–6 TeV can still give an EW fine-tuning of about ${\sim }100$, for the low $\tan \beta$ regime considered here.

The case of constrained “non-linear” MSSM at small $\tan \beta \le 10$, for which we found “acceptable” values for $\Delta _{m,q}$, is the most conservative scenario. We saw in Figs. 2 and 3 that for the same $m_h$ a larger $\tan \beta$ reduces fine-tuning and this behavior continues to $\tan \beta \sim 40$–$50$. Then additional Yukawa couplings also play a significant role at larger $\tan \beta$ and reduce fine-tuning further by improving the radiative EW symmetry breaking for the same $m_h$ (this is because radiative EW symmetry breaking effects are enhanced relative to opposite, QCD ones that increase fine-tuning [38–41]). We thus expect that for the case of large $\tan \beta$ with additional Yukawa couplings included the values quoted here for $\Delta _m$, $\Delta _q$ be maintained or reduced further.

Unlike other attempts to reduce the EW fine-tuning, the present case has the advantage that it does not introduce new states in the visible sector. However, there still is a “cost” at the phenomenological level. In models with a TeV scale for SUSY breaking, the gravitino is very light (milli-eV) and the usual MSSM-like account for dark matter (as due to the LSP) cannot apply. This is a standard problem for models with a low scale of SUSY breaking, and alternative dark matter candidates need to be considered (the axino [76], or the axion [77]; for a review see [78]).

## Conclusions

The significant amount of EW fine-tuning $\Delta$ present in the MSSM-like models for $m_h\approx 126$ GeV has prompted an increased interest in finding ways to reduce its value. This is motivated by the fact that $\Delta$ is usually regarded as a measure of the success of SUSY in solving the hierarchy problem. Additional reasons to seek a low $\Delta$ exist, from the relation of the EW fine-tuning to the variation $\delta \chi ^2$ about the minimal chi-square $\chi ^2_\mathrm{min}$ and the s-standard deviation upper bound on $\delta \chi ^2$ usually sought in the data fits. Reducing $\Delta$ can indeed be achieved, but it usually requires the introduction of additional fields in the visible sector, beyond those of the original model. For example, one can consider MSSM-like models with additional, massive gauge singlets present, extra gauge symmetries, etc.

Another point of view is that a large EW fine-tuning may indicate a problem with our understanding of supersymmetry breaking. Motivated by this we considered the case of MSSM-like models with a low scale of supersymmetry breaking in the hidden sector, $\sqrt{f}\sim$ few TeV. As a result of this, sizeable quartic effective interactions are present in the Higgs potential, generated by the exchange of the auxiliary field of the goldstino superfield. Such couplings are proportional to the ratio of the soft breaking terms $m_\mathrm{soft}$ in the visible sector to the SUSY breaking scale $\sqrt{f}$ of the hidden sector. Thus, such couplings are significant in models with $\sqrt{f} \sim$ few TeV and are negligible when $\sqrt{f}$ is large, which is the usual MSSM scenario. These couplings have significant implications for the Higgs mass and the EW fine-tuning. This behavior is generic in low-scale SUSY models.

For the most conservative case of a constrained “non-linear” MSSM model and at low $$\tan \beta $$, we computed the level of EW scale fine-tuning measured by two definitions for $$\Delta $$ ($$\Delta _m$$, $$\Delta _q$$). We examined $$\Delta _{m,q}$$ as a function of the SM-like Higgs mass, in the one-loop approximation for these quantities. The results show that for $$m_h\approx 126$$ GeV, fine-tuning is reduced from *minimal* values of $${\approx }800$$–$$1000$$ in the constrained MSSM to more acceptable values of $${\sim }80$$–$$100$$ in our model with $$\sqrt{f}\sim 2.8 $$–$$ 3.2$$ TeV. These values for $$\Delta $$ are expected to be further reduced by considering non-universal gaugino masses. We argued that a similar reduction of $$\Delta $$ is expected at large $$\tan \beta $$ in our model. For larger $$\sqrt{f}$$, usually above $$10$$ TeV, one recovers the case of MSSM-like models. Unlike other similar studies, the reduction of $$\Delta $$ was possible *without* additional fields in the visible sector and depends only on the ratio(s) $$m_\mathrm{soft}^2/f$$. One may consider the intriguing possibility of increasing *simultaneously* one of the soft masses $$m_\mathrm{soft}$$ (say $$m_0$$) and $$\sqrt{f}$$, with their ratio fixed (this could keep unchanged the leading corrections $$\mathcal{O}[(m_\mathrm{soft}^2/f)^2]$$ for the Higgs mass and $$\Delta $$). This is relevant if no superpartners are found near the TeV scale.

We assumed that in our case the sgoldstino was massive enough and integrated out, by using the superfield constraint that decouples it from the low energy. Corrections to our result can then arise from the scalar potential for the sgoldstino that depends on the structure of its Kähler potential (which gives mass to it) and the superpotential in the hidden sector. Another correction can arise from future experimental constraints that may increase the lower bounds on the value of $\sqrt{f}$, currently near ${\approx }700$ GeV, if no supersymmetry or other new physics signal is found.

## Appendix

The coefficients $\sigma _i$ at the EW scale, used in the text, Eq. (28), have the following values

A.1 $$\begin{aligned}&\sigma _1(t_z)=0.532, \sigma _2(t_z)=0.282(4.127h_t^2-2.783)\nonumber \\&\quad (1.310-h_t^2)^{1/4},\nonumber \\&\sigma _3(t_z)= -0.501h_t^2(1.310-h_t^2)^{1/4},\nonumber \\&\quad {\sigma }_4(t_z)=0.532-5.233h_t^2+1.569h_t^4,\nonumber \\&{\sigma }_5(t_z)= 0.125h_t^2(10.852h_t^2-14.221),\nonumber \\&\quad {\sigma }_6(t_z)=-0.027h_t^2(10.852\,h_t^2-14.221),\nonumber \\&{\sigma }_7(t_z)=1-1.145 h_t^2, {\sigma }_8(t_z)= 1.314 (1.310-h_t^2)^{1/4} \end{aligned}$$

where $h_t$ is evaluated at $m_Z$ and $m_t=h_t(t_{m_t})(v/\sqrt{2})\sin \beta$ ($v=246$ GeV), $t=\ln \Lambda ^2/q^2$, $t_z=\ln \Lambda ^2_{UV}/m_Z^2$.

## Footnotes

1 Hence the name of the model: “non-linear” MSSM.

2 We stress that at energy scales below $m_\mathrm{soft}$, similar constraints to that used for $X$ ($X^2=0$) can be applied to the MSSM superfields themselves and correspond to integrating out the massive superpartners [8, 9].

3 In the standard notation for a two-Higgs doublet model $V=\tilde{m}_1^2\vert h_1\vert ^2 +\tilde{m}_2^2\vert h_2\vert ^2 -(m_3^2 h_1 \cdot h_2+\hbox {h.c.})+ \frac{1}{2}\lambda _1 \vert h_1\vert ^4 +\frac{1}{2}\lambda _2 \vert h_2\vert ^4 +\lambda _3 \vert h_1\vert ^2\vert h_2\vert ^2+\lambda _4\vert h_1\cdot h_2\vert ^2 + \Big [ \frac{1}{2}\lambda _5(h_1\cdot h_2)^2+\lambda _6\vert h_1\vert ^2 (h_1 \cdot h_2)+ \lambda _7\vert h_2\vert ^2 (h_1 \cdot h_2)+\hbox {h.c.}\Big ]$ where $\tilde{m}_1^2=m_1^2+\vert \mu \vert ^2$, $\tilde{m}_2^2=m_2^2+\vert \mu \vert ^2$. $\lambda _1/2=g^2/8+m_1^4/f^2$, $\lambda _2/2=g^2 (1+\delta )/8+m_2^4/f^2$, $\lambda _3=(g_2^2-g_1^{2})/4+2 m_1^2 m_2^2/f^2$, $\lambda _4= -g_2^2/2+m_3^4/f^2$, $\lambda _5=0$, $\lambda _6=-m_1^2 m_3^2/f^2$, $\lambda _7=-m_2^2 m_3^2/f^2$, $g^2=g_1^2+g_2^2$.

4 Effective operators in the Higgs sector in the SUSY context were discussed in the past [49, 62–69].

5 More exactly $A_t=A_u/m_0$ with $A_u$ as in Eq. (7).

6 Also $\lambda \equiv (\lambda _1/2) c_\beta ^4+ (\lambda _2/2)s_\beta ^4 +(\lambda _3+\lambda _4+\lambda _5)s_\beta ^2c_\beta ^2 +2\lambda _6c_\beta ^3s_\beta + 2\lambda _7c_\beta s_\beta ^3$ where we used the notation of footnote 3 and $s_\beta =\sin \beta$, $c_\beta =\cos \beta$, $u\equiv \tan \beta =v_2/v_1$, $h_i=1/\sqrt{2}(v_i+\tilde{h}_i)$, $m_Z^2=(g_1^2+g_2^2)v^2/4$.

7 As we shall detail shortly for the case of the constrained MSSM.

8 For this exponential dependence on $m_h$ see figures 1 and 6 in the first reference in [38–41].

9 As we show shortly for the conservative case of the constrained “non-linear” MSSM, at small $\tan \beta$, fine-tuning is already acceptable, thus at larger $\tan \beta$ $\Delta$ is expected to be similar or further reduced.

10 For $m_h\approx 126$ GeV, in constrained MSSM $\Delta _{m,q}\sim 800$–$1000$ [30].

11 This is a conservative bound, since in the potential (Eq. (9)) and in the Higgs mass of Eqs. (18), (20) the leading corrections are actually of the (higher) order $\mathcal{O}(m_\mathrm{soft}^4/f^2)$ (we ignore the $\mathcal{O}(m_\mathrm{soft}^6/f^3)\le (1/4)^3\sim 1.5~\%$ or about $2$ GeV to the Higgs mass). Similar for the fine-tuning $\Delta$; see for example Eq. (29) or the exact results in Sect. 4.2.2, where the leading terms are $\mathcal{O}(1/f^2)$.
